# Correlation Between C-MYC, BCL-2, and BCL-6 Protein Expression and Gene Translocation as Biomarkers in Diagnosis and Prognosis of Diffuse Large B-cell Lymphoma

**DOI:** 10.3389/fphar.2018.01497

**Published:** 2019-01-07

**Authors:** YunXiang Zhang, Hui Wang, Cuiai Ren, Hai Yu, Wenjia Fang, Na Zhang, Sumei Gao, Qian Hou

**Affiliations:** ^1^Department of Pathology, Weifang People's Hospital, Weifang, China; ^2^Department of Pathology, Werfang Traditional Chinese Hospital, Weifang, China; ^3^Department of Clinical Medicine, Nanchang University Medical College, Nanchang, China

**Keywords:** diffuse lange B-cell lymphoma, C-MYC, BCL-2, BCL-6, gene translocation, gene amplification

## Abstract

This study investigates the protein expression of C-MYC, BCL-2, and BCL-6 in diffuse large B-cell lymphoma (DLBCL) and their relationship with genetic abnormalities. A retrospective study of 42 cases on paraffin-embedded tissue specimens diagnosed with DLBCL was performed using immunohistochemistry (IHC) and fluorescence *in situ* hybridization (FISH). The expression of C-MYC, BCL-2, BCL-6 protein, and gene abnormalities in these tissue samples was analyzed. The relationship in genetic abnormalities and Ki-67, Hans classification, gender, and age was also evaluated. It was found that the positive rate of C-MYC expression was 47.6% (20/42), the rate of C-MYC gene abnormality was 26.2% (11/42), in which gene translocation accounted for 23.8% (10/42) and gene amplification 2.4% (1/42); C-MYC protein expression was positively correlated with C-MYC gene translocation (χ^2^ = 11.813; *P* = 0.001); C-MYC gene translocation was mainly found in germinal center B cell type (χ^2^ = 4.029; *P* = 0.045). The positive rate of BCL-2 protein expression was 85.71% (36/42), the positive rate of translocation was 42.86% (18/42) and the amplification rate was 26.19% (11/42); the overexpression of BCL-2 protein was correlated with the BCL-2 translocation (χ^2^ = 3.407; *P* = 0.029). The positive rate of BCL-6 protein expression was 45.24% (19/42), the positive rate of BCL-6 translocation was 14.29% (6/42) and the positive rate of BCL-6 amplification was 7.14% (3/42); the overexpression of BCL-6 protein was significantly correlated with BCL-6 translocation (χ^2^ = 6.091; *P* = 0.014). The Ki-67 index was significantly higher in C-MYC translocation cases than in non-C-MYC translocation cases (χ2 = 4.492; P = 0.034). Taken together, our results suggest that the protein expression of C-MYC, BCL-2, and BCL-6 are positively correlated with their gene translocation. Overexpression of C-MYC, BCL-2, BCL-6 protein suggests the possibility of translocation. Therefore, immunohistochemical detection of C-MYC, BCL-2, and BCL-6 are useful in diagnosis and prognosis of DLBCL.

## Introduction

Diffuse large B-cell lymphoma (DLBCL) is a highly heterogeneous lymphoid hematopoietic malignancy which is one of the most common types of adult non-Hodgkin's lymphoma (NHL). The incidence of DLBCL is about 30% in Europe and the United States (Chung and Levens, 2005), while in China, it is as high as 40% of all lymphomas (Li et al., 2012). DLBCL can be divided into germinal center B cell type (GCB type) and non-germinal center B cell type (non-GCB type) based on Hans analysis (Hans et al., 2004). Hans criteria is based on the presence or absence of three biomarkers, CD10, BCL-6, and MUM-1in immunohistochemical staining using antibodies against CD10, IRF4/MUM1, and BCL6. Those with the number of CD10 positive cells greater than 30%, as well as BCL6 positive belong to the germinal center B cell subtype, and the rest are judged as non-germinal center B cell subtype (Hans et al., 2004). Different types of lymphoma have differences in cell morphology, protein expression, genetic changes, and therapeutic responsiveness. Therefore, understanding the molecular characteristics of lymphoma are critical for individualized patient care.

In patients with DLBCL, anthracycline chemotherapy can achieve 70% of remission. However, only 50–60% of patients achieve long-term disease-free survival. Most of the unresolved patients are highly aggressive and have chemotherapy resistance to first-line regimens. The combination of rituximab and other chemotherapeutic drugs has greatly improved the response rate and prognosis of DLBCL patients. It would be greatly helpful to subgrouping DLBCL, to understand its pathogenesis and to guide clinical drug use.

In recent years, with the progress of molecular genetic research, it was understood that abnormal expression of some genes is directly correlated with the occurrence, development, treatment response and prognosis of DLBCL. Previous study indicated that some lymphoma is highly aggressive when it expressed C-MYC gene accompanied by expression of BCL-2 or BCL-6 genes (Valera et al., 2013). In 2016, the guidelines of the National Comprehensive Cancer Network (NCCN) recommended using FISH to detect gene abnormality, such as translocation in lymphoma cells.

A B-cell lymphoma with a combination of C-MYC and BCL-2 or BCL-6 translocations is called a Double-Hit lymphoma (DHL), and the one with three gene translocations is called a Triple-Hit lymphoma (THL) (Campo et al., 2011). In other words, DHL/THL refers to a B cell lymphoma with multiple activated oncogenes. C-MYC gene is the most common mutated gene in lymphomas. C-MYC and BCL-2 simultaneous translocation is the most common DHL, while C-MYC/BCL-6 DHL and C-MYC/BCL-2/BCL-6 THL are rare. The 2016 version of the WHO lymphoma classification suggests that C-MYC genetic alteration is one of the important diagnostic indicators of DLBCL, and defines DHL and THL as high-grade B-cell lymphoma (Arber et al., 2016). The understanding of DHL and THL is important because it may change the clinical management and prognosis of lymphomas. Therefore, further identifying the genetic abnormalities of C-MYC, BCL-2, and BCL-6 in DLBCL is of great significance to guide clinical diagnosis and treatment of DLBCL.

In the present study, we use immunohistochemistry (IHC) and fluorescence *in situ* hybridization (FISH) to detect the expression and genetic abnormalities, particularly gene translocation and amplification of C-MYC, BCL-2, and BCL-6 genes in patients with DLBCL. We further analyze their correlation with clinical characteristics, to provide guidelines for using these biomarkers for diagnosing, guiding treatment and assessing prognosis of DLBCL.

## Materials and Methods

### Patients and Specimens

This study was approved by the Ethics Committee of Weifang People's Hospital. A total of 67 cases of paraffin-embedded tissue specimens from patients who were diagnosed with DLBCL for the first time in the Department of Pathology, Weifang People's Hospital from January 2015 to October 2016 were collected. According to the diagnostic criteria of 2016 WHO classification of hematopoietic and lymphoid tissue tumors (Arber et al., 2016), two senior pathologists in the Department of Pathology of Weifang People's Hospital reviewed the cases, and 42 cases were eventually included in the study (25 patients were excluded due to incomplete clinical data and inaccurate classification). Complete pathological data and patients' informed consent were obtained. There were 26 males and 16 females with an average age of 58.9 ± 12.3 years old (range 43–80 years old). Specimens were from various sources: 27 cases of superficial lymph nodes, 6 cases of subcutaneous soft tissue, 6 cases of gastrointestinal tract, 1 case of mesentery and spleen, and 1 case of tonsil. Among these specimens, 16 cases were of GCB type and 26 cases of non-GCB type, Another 20 patients with reactive hyperplastic superficial lymph nodes were used as negative controls, including 12 males and 8 females, with an average age of 58.0 ± 10.5 (range 41–77 years). All specimens were fixed in 10% neutral formalin. Conventional paraffin embedding was performed. These specimens were assembled into a tissue microarray with the core diameter of 3 mm.

### Immunohistochemistry

Immunohistochemistry was performed using Roche Benchmark XT Ventana automatic immunohistochemical staining instrument. The staining procedure was set up strictly according to the instructions for automatic immunohistochemical detection. A pair of positive and negative tissues was placed in each tissue microarray as required. BCL-2 and C-MYC rabbit anti-human monoclonal antibody (clone number EP36, and EP121), and BCL-6 mouse anti-human monoclonal antibody (clone number LN22) were purchased from Beijing Zhongshan Jinqiao Biotechnology Co., Ltd.

### Fluorescence *in situ* Hybridization (FISH)

FISH detection was performed using 3 μm paraffin tissue sections. C-MYC, BCL-2, and BCL-6 probes were purchased from Beijing Jinpujia Company. The C-MYC normal signal is a yellow signal from fused red and green. The C-MYC gene translocation is considered as positive when a yellow signal, a green signal, and a red signal, or two red signals or two green signals appear in the nucleus. The C-MYC gene amplification is interpreted as positive when three or more red-green fusions appeared as yellow signals were observed.

Analysis and determination of BCL-2 and BCL-6 test results: The BCL-2 and BCL-6 probes are a two-color fusion probe, and normally two red and two green signals are separated from each other. When a red signal, a green signal, and a red-green fused yellow signal or two yellow signals appear, it was interpreted as a positive BCL-2 or BCL-6 gene translocation. When three or more red or green signals appear in the nucleus, it was considered as positive BCL-2 or BCL-6 gene amplification.

### Establishing the Staining Analyzing Threshold

Twenty non-lymphoma (reactive hyperplastic lymph nodes) tissue specimens were randomly selected as the control group. FISH detection of BCL-2, BCL-6, and C-MYC genes was performed. 200 cells per sample were analyzed and the number of cells, the mean percentage value and standard deviation of abnormal signals were calculated. The positive threshold was determined to be mean percentage ± 3 standard deviation. The results were analyzed based on the threshold. The number of translocation and the number of abnormal amplified signal of cells in the lymphoma and non-lymphoma specimens were counted, and the average value was taken. The abnormality threshold was used to judge the detection result: the result was judged as negative if the percentage of abnormal signal cells was less than the threshold; the result was judged as positive if the percentage of abnormal signal cells was greater than the threshold. If the percentage of abnormal signal cells was equal to the threshold value, then the number of cells of the test sample was increased to obtain a final result.

### Statistical Analysis

SPSS 20.0 software was applied for statistical analysis. The differences between the factors were analyzed by chi-square test. When the conditions of chi-square test cannot be met, Fisher's exact probability method was used. *P* < 0.05 was considered statistically significant.

## Results

### The Morphology of DLBCL

**Figure 1:**
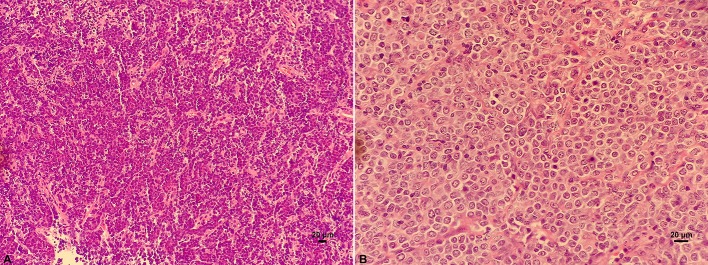
HE staining on the structure of lymph nodes of DLBCL. **(A)** Diffuse hyperplasia of lymphocytes. **(B)** Higher magnification showing the morphology of atypical and blast DLBCL cells.

Under the light microscope, DLBCL tumor tissue was seen as diffuse hyperplasia of large B lymphocytes that replaced normal lymph node structure. The morphology of these lymphocytes was atypical. The cell volume is more than twice as that of conventional cells, and nucleus of the tumor cells is mostly larger than that of normal lymphocytes (Figure 1).

### Immunohistochemical Staining

**Figure 2:**
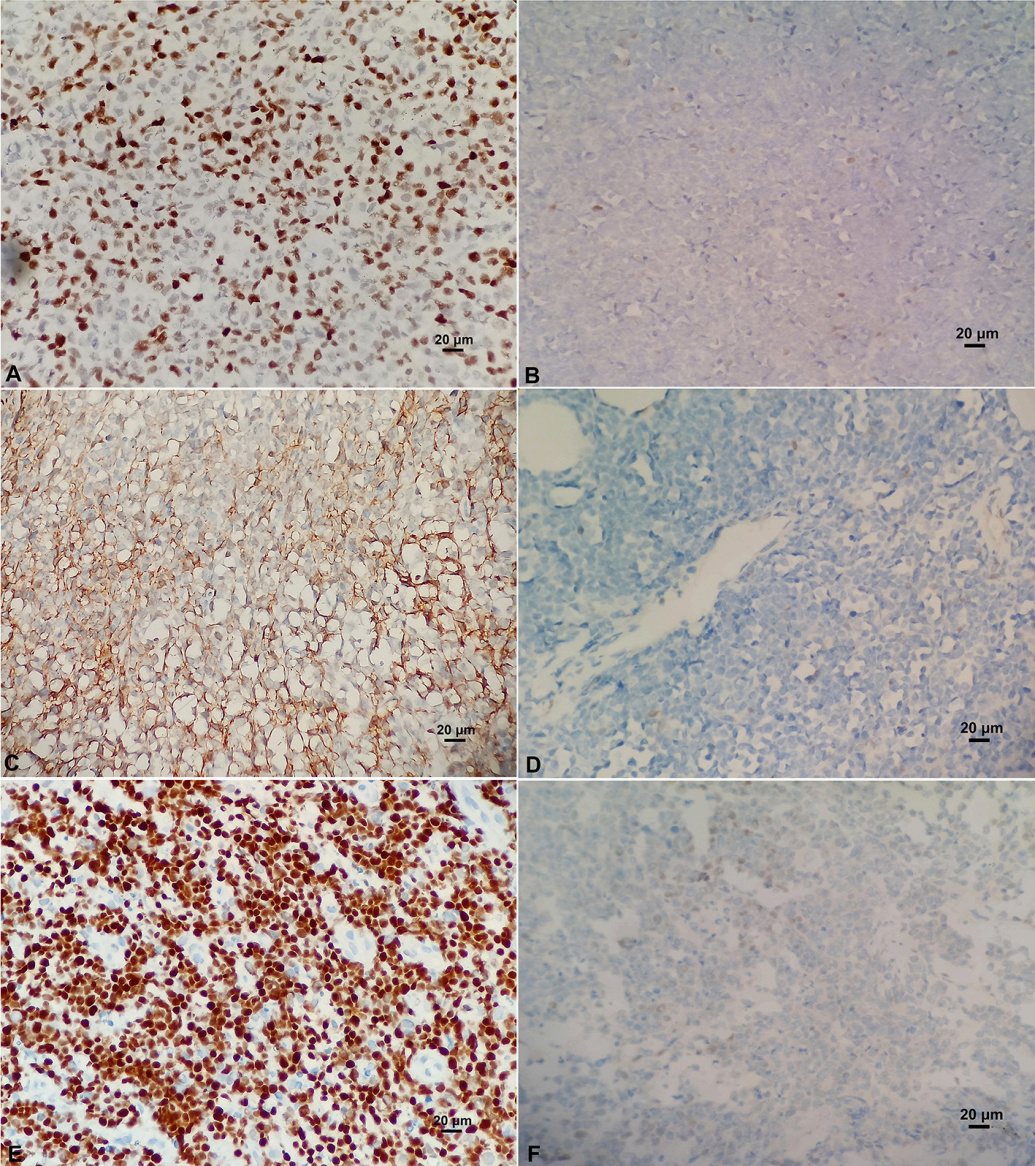
**(A)** Positive C-MYC protein expression (×400). **(B)** Negative C-MYC protein expression (×400). **(C)** Positive BCL-2 protein expression (×400). **(D)** Negative BCL-2 protein expression (×400). **(E)** Positive BCL-6 protein expression (×400). **(F)** Negative BCL-6 protein expression (×400).

BCL-6 and C-MYC positive staining were located in the nucleus, while BCL-2 positive staining was in the cytoplasm (Figure 2). According to previous reports, the staining could be judged as positive if the number of tumor cells positive for C-MYC ≥ 40%, BCL-2 ≥ 50%, or BCL-6 ≥ 30% of tumor tissues (Hans et al., 2004; Horn et al., 2013). In our study, the positive rate of immunohistochemical staining of C-MYC, BCL-2, and BCL-6 protein was 47.6% (20/42), 85.71% (36/42), and 45.24% (19/42), respectively. Based on the Hans classification, the ratio of non-GCB type was 61.9% (26/42), which was higher than that of GCB type (38.1%, 16/42) in these 42 cases. The difference between GCB and non- GCB types was not statistically significant (*P* > 0.05). Similarly, there was no significant difference in the distribution of C-MYC, BCL-2, and BCL-6 protein expression in Hans classification, gender, and age (Table 1).

**Table 1:** Association of C-MYC, BCL-2, and BCL-6 protein expression and clinical characteristics.

		**Hans typing**		**Gender**		**Age**		**Total cases**
		**GCB**	**Non-GCB**	**Male**	**Female**	**≥50 y**	**< 50 y**
C-MYC expression	Positive	8	12	15	5	17	3	20
	Negative	8	14	11	11	16	6	22
χ^2^		0.059		0.776		0.35		
P		0.808		0.096		0.554		
BCL-6	Positive	12	24	21	15	27	9	36
expression	Negative	4	2	5	1	6	0	6
χ^2^		1.216		0.59		0.713		
P		0.27		0.476		0.167		
BCL-2 expression	Positive	7	12	12	7	16	3	19
	Negative	9	14	14	9	17	6	23
χ^2^		0.023		0.023		0.186		
P		0.879		0.879		0.666		
C-MYC/BCL-2 co-expression	Positive	5	10	11	4	12	3	15
	Negative	11	16	15	12	21	6	27
χ^2^		0.224		1.292		0		
P		0.636		0.256		1		

Among these 42 cases of DLBCL, 15 cases (35.71%, 15/42) were co-expressed with C-MYC and BCL-2 or BCL-6, 8 cases (19.05%, 8/42) were C-MYC and BCL-2 double positive expression, 7 patients (16.67%, 7/42) had C-MYC, BCL-2, and BCL-6 triple positive expression, and no cases of C-MYC and BCL-6 double expression were found. Although among these 15 cases of C-MYC, BCL-2, and BCL-6 protein co-expression, there were 10 cases of non-GCB type (38.46%, 10/26), 11 cases of men (42.31%, 11/26), 12 cases are ≥50 years old (36.36%, 12/33), which made up a high proportion of Hans classification, gender, and age grouping, but these differences were not statistically significant (Table 1).

### FISH Test

#### C-MYC Gene Abnormality

**Figure 3:**
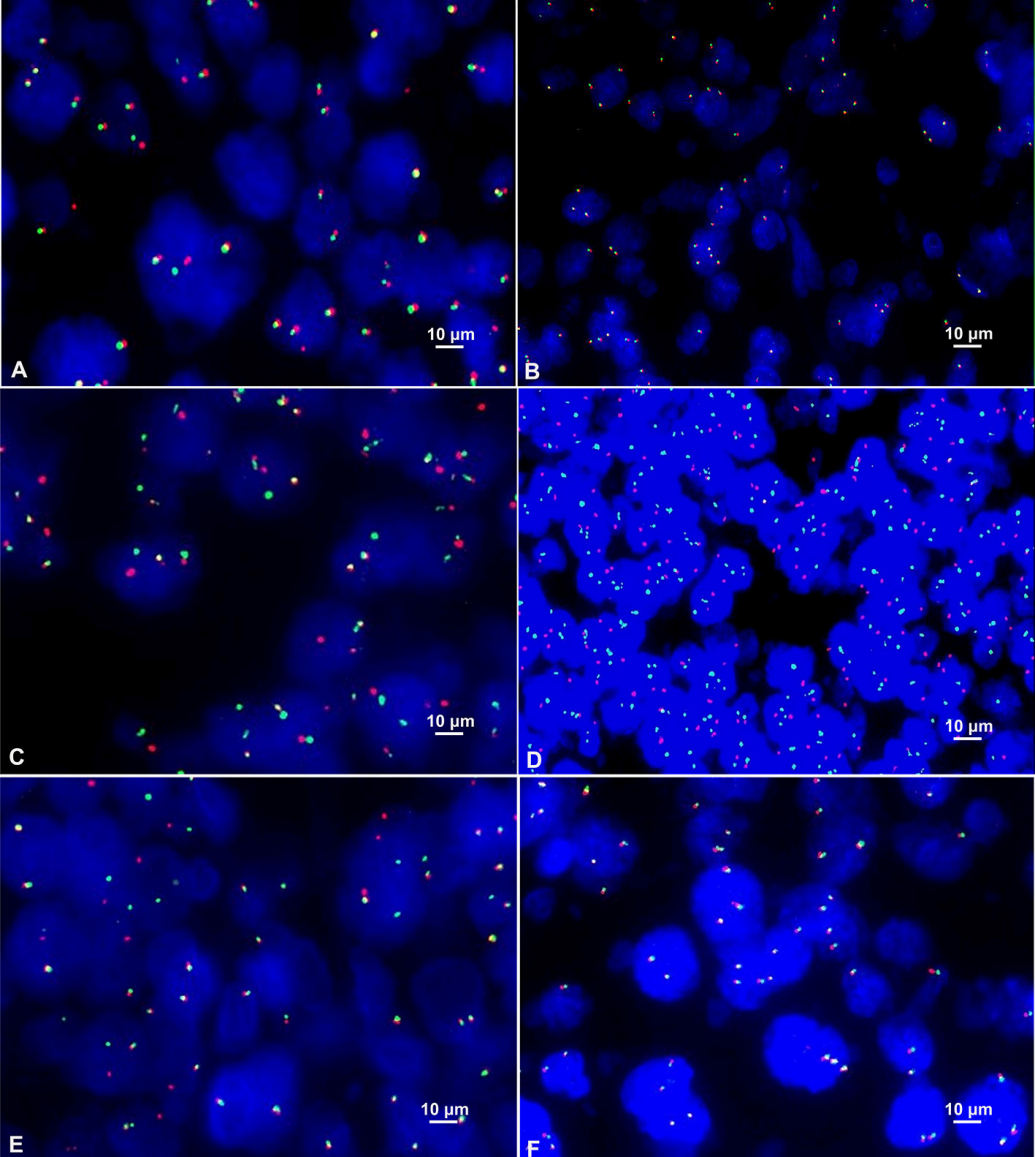
**(A)** Positive C-MYC gene translocation showed 1 red signal, 1 green signal, and 1 red-green-fused yellow signal in the nucleus (FISH×1000). **(B)** Positive C-MYC gene amplification showed ≥3 red-green fused yellow signals in the nucleus (FISH×1000). **(C)** Positive BCL-2 gene translocation showed 1 red signal, 1 green signal and 1 red-green-fused yellow signal in the nucleus (FISH×1000). **(D)** Positive BCL-2 gene amplification showed ≥3 red or green signals in the nucleus (FISH×1000). **(E)** Positive BCL-6 gene translocation showed 1 red signal, 1 green signal and 1 red-green-fused yellow signal in the nucleus (FISH×1000). (**F)** Positive BCL-6 gene amplification showed ≥3 red-green-fused yellow signals in the nucleus (FISH×1000).

Among the 42 cases of DLBCL, C-MYC translocation was detected in 10 cases (23.8%) (Figure 3A). All of these cases were positive for C-MYC protein expression. No case of C-MYC translocation was found in negative C-MYC protein expression group. The difference between the positive and negative C-MYC translocation was statistically significant (*P* = 0.001, Table 2). There were 43.8% (7/16) of GCB type and 11.5% (3/26) of non-GCB type had C-MYC translocation, and the difference between the two groups had statistical significance (*P* = 0.045). Among the C-MYC translocation cases, 8 were male (8/26, 30.8%) and 2 were female (2/16); 10 patients with C-MYC translocation were ≥50 years old (10/33, 30.30%). The difference of C-MYC translocation in different age and gender groups were not statistically significant (*P* > 0.05) (Table 2). These results suggested that C-MYC translocation was associated with C-MYC protein expression and GCB type, but not associated with age and gender.

**Table 2:** Association of C-MYC protein expression and gene translocation, amplification, and clinical characteristics.

**Indicator**		**Total cases**	**C-MYC translocation**		**χ^**2**^**	***P***	**C-MYC amplification**		**χ2**	***P***
			**Positive**	**Negative**			**Positive**	**Negative**		
C-MYC expression	Positive	20	10	10	11.813	0.001	0	20	0.000	1.000
	Negative	22	0	22			1	21		
Hans typing	GCB type	16	7	9	4.029	0.045	0	16	0.000	1.000
	Non-GCB type	26	3	23			1	25		
Gender	Male	26	8	18	0.954	0.329	0	26	0.000	0.381
	Female	16	2	14			1	15		
Age	≥50 years	33	10	23	2.104	0.147	1	32	0.000	1.000
	< 50 years	9	0	9			0	9		

Among the 42 cases of DLBCL, only one case (2.4%) of C-MYC amplification was detected (Figure 3B). There was no statistical significance concerning the differences in C-MYC amplification among C-MYC protein expression, Hans classification, gender, and age (*P* > 0.05, Table 2).

#### BCL-2 Gene Abnormality

In the FISH test, 18 cases of BCL-2 translocation were found (Figure 3C), all of which had positive BCL-2 protein expression, while no cases of BCL-2 translocation occurred in negative BCL-2 protein expression group. The difference in BCL-2 translocation between BCL-2 protein expression and non-BCL-2 protein expression groups was statistically significant (*P* = 0.029, Table 3). Among the cases of positive BCL-2 translocation, 7 cases (7/16, 43.75%) were GCB and 11 cases (11/26, 42.31%) were non-GCB type. There was no significant difference between the two groups (*P* = 0.927, Table 3). There were 10 males (10/26, 38.46%) and 8 females (8/16, 50%); 13 cases (13/33, 39.39%) were ≥ 50 years old, and 5 cases were < 50 years old (5/9, 55.56%). There was no significant difference in either age or gender for BCL-2 gene translocation (*P* > 0.05, Table 3). These results suggested that BCL-2 translocation was associated with BCL-2 protein expression, but not associated with GCB type, age, and gender.

**Table 3:** Association of BCL-2 protein expression, gene translocation, and amplification and clinical characteristics.

**Indicator**		**Total cases**	**BCL-2 translocation**		**χ^**2**^**	***P***	**BCL-2 amplification**		**χ^**2**^**	***P***
			**Positive**	**Negative**			**Positive**	**Negative**		
BCL-2 expression	Positive	36	18	18	3.407	0.029	11	25	1.155	0.283
	Negative	6	0	6			0	6		
Hans typing	GCB type	16	7	9	0.008	0.927	5	11	0.050	0.823
	Non-GCB type	26	11	15			6	20		
Gender	Male	26	10	16	0.538	0.463	6	20	0.050	0.823
	Female	16	8	8			5	11		
Age	≥50 years	33	13	20	0.239	0.625	8	25	0.015	0.903
	< 50 years	9	5	4			3	6		
Total cases		42	18	24			11	31		
Total cases		42	10	32			1	41		

BCL-2 amplification was detected in 11 cases (26.19%) (Figure 3D). These cases were positive for BCL-2 protein expression. However, there was no correlation between BCL-2 protein expression and BCL-2 gene amplification (*P* = 0.283, Table 3), neither was there significant difference in BCL-2 amplification in Hans classification, gender, and age by statistical analysis (*P* > 0.05, Table 3).

#### BCL-6 Gene Abnormalities

Among these 42 cases of DLBCL, BCL-6 translocation was detected in 6 cases (14.29%) (Figure 3E), all of which were positive for BCL-6 protein expression, and BCL-6 translocation did not occur in negative BCL-6 protein expression group. The difference in BCL-6 translocation between positive and negative BCL-6 protein expression groups was statistically significant (*P* = 0.014, Table 4). BCL-6 translocation was found in 12.50% (2/16) GCB type and 15.38% (4/26) non-GCB type, and the difference between the two groups was not statistically significant (*P* = 1.000, Table 4). Although 6 patients with BCL-6 translocation were men and aged 50 years or older, there was no significant difference in either age or gender for BCL-6 translocation (*P* > 0.05, Table 4). These results suggested that BCL-6 translocation was associated with BCL-6 protein expression, but not associated with GCB type, age, or gender.

**Table 4:** Association of BCL-6 protein expression, gene translocation and amplification, and clinical characteristics.

**Indicator**		**Total cases**	**BCL-6 translocation**		**χ^**2**^**	***P***	**BCL-6 amplification**		**χ2**	**P**
			**Positive**	**Negative**			**Positive**	**Negative**		
BCL-6 expression	Positive	19	6	13	6.091	0.014	1	18	0.000	1.000
	Negative	23	0	23			2	21		
Hans typing	GCB type	16	2	14	0.000	1.000	1	15	0.000	1.000
	Non-GCB type	26	4	22			2	24		
Gender	Male	26	6	20	2.629	0.105	1	25	0.194	0.659
	Female	16	0	16			2	14		
Age	≥50 years	33	6	27	0.713	0.398	1	32	0.000	0.111
	< 50years	9	0	9			2	7		
Total cases		42	18	24			3	39		

BCL-6 amplification was detected in 3 cases (7.14%) (Figure 3F), of which 1 case was positive for BCL-6 protein (5.26%, 1/19), and the other 2 cases were negative for BCL-6 protein (8.70%, 2/23). There was 1 case of GCB type (6.25%, 1/16) and 2 cases of non-GCB type (7.69%, 2/26); 1 case of male (3.85%, 1/26), 2 cases of female (12.50%, 2/16); 1 patient (3.03 %, 1/33) with age ≥ 50 years and 2 patients (22.22%, 2/9) with age < 50 years. There was no significant difference in BCL-6 amplification among BCL-6 protein expression, Hans classification, gender, or age (*P* > 0.05, Table 4).

#### Double-Hit Lymphomas (DHL)

Among these 42 cases of DLBCL, only 2 cases (4.76%) of DHL were detected, one had both C-MYC and BCL-2 translocation and is non-GCB type; the other was both C-MYC gene and BCL-6 gene translocation, and is GCB type. Although both cases of DHL are older than 50 years old with double expression of C-MYC and BCL-2 protein, meaning that they were dual expressor of lymphomas (DEL), analysis had suggested that there was no significant correlation between DHL and DEL, Hans classification, gender or age (*P* > 0.05, Table 5).

**Table 5:** The relationship between DHL and clinical characteristics.

**Indicator**		**Total cases**	**DHL**		**χ^**2**^**	***P***
			**Positive**	**Negative**		
DEL	Positive	15	2	13	1.412	0.235
	Negative	27	0	27		
Hans typing	GCB type	16	1	15	0.000	1.000
	Non-GCB type	26	1	25		
Gender	Male	26	2	24	0.153	0.696
	Female	16	0	16		
Age	≥50 years	33	2	31	0.000	1.000
	< 50 years	9	0	9		
Total cases		42	2	40		

### Ki-67 Proliferation Index and C-MYC, BCL-2, BCL-6 Gene Abnormalities

**Figure 4:**
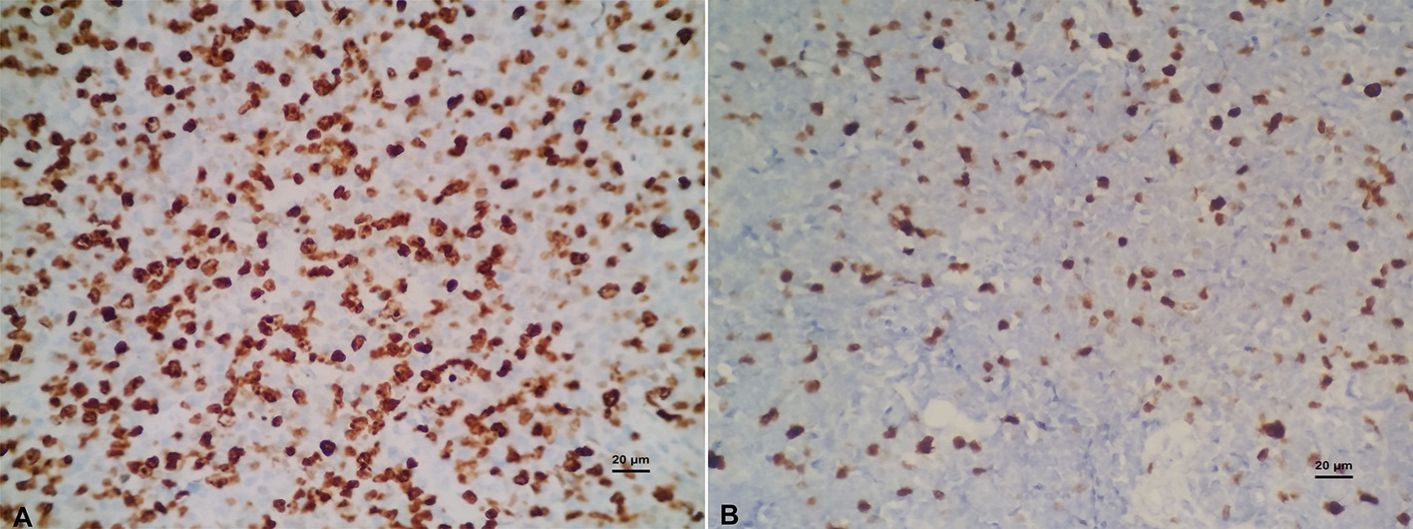
**(A)** 90% Ki-67 protein expression (×400). **(B)** 35% Ki-67 protein expression (×400).

Ki-67 immunohistochemical staining was used to examine the proliferative ability of lymphoma tissues. The overexpression of Ki-67 in DLBCL indicates that the tumor is invasive, rapidly progress, and has poor clinical prognosis. The Ki-67 immunohistochemical staining was performed and intensity was quantified in all specimens (Figure 4). The cut-off value of Ki-67 positivity was set to 80 and 90%. According to group statistical analysis, 12 cases (28.57%, 12/42) had positive Ki-67 ≥ 90%, 25 cases (28.57%, 25/42) were Ki-67 ≥ 80% in 42 cases of DLBCL. Six of Ten cases of C-MYC translocation cases were Ki-67 ≥ 90, 60, and 18.75% (6/32) of non-C-MYC translocation cases were Ki-67 ≥ 90%. There were significant differences of Ki-67 positivity in C-MYC and non-C-MYC translocation cases (*P* < 0.05, Table 6). Ki-67 was 90 and 70% in 2 cases of DHL, and 90% in 1 case of C-MYC amplification. BCL-2 and BCL-6 translocation or amplification with overexpression of Ki-67 were analyzed, and it was found that there was no significant correlation in Ki-67 overexpression and C-MYC amplification (*P* > 0.05, Table 6). This result indicated that C-MYC translocation is more useful than C-MYC amplification in terms of diagnosis and prognosis of DLBCL.

**Table 6:** The relationship between Ki-67 and C-MYC BCL-2, and BCL-6 gene abnormalities.

**Types of gene abnormalities**		**Total cases**	**Ki-67**		**χ^**2**^**	***P***	**Ki-67**		**χ^**2**^**	***P***
			**≥ 90% cases**	**< 90% cases**			**≥ 80% cases**	**< 80% cases**		
C-MYC translocation	Positive	10	6	4	4.492	0.034	7	3	0.163	0.686
	Negative	32	6	26			18	14		
C-MYC amplification	Positive	1	1	0	0.230	0.631	1	0	0.000	1.000
	Negative	41	11	30			24	17		
BCL-2 translocation	Positive	18	3	15	2.188	0.139	9	9	1.186	0.276
	Negative	24	9	15			16	8		
BCL-2 amplification	Positive	11	1	10	1.629	0.202	5	6	0.561	0.454
	Negative	31	11	20			20	11		
BCL-6 translocation	Positive	6	1	5	0.044	0.834	5	1	0.696	0.404
	Negative	36	11	25			20	16		
BCL-6 amplification	Positive	3	1	2	0.000	1.000	1	2	0.122	0.727
	Negative	39	11	28			24	15		
Total cases		42	12	30			25	17		

## Discussion

### Characteristics of Diffuse Large B-Cell Lymphoma

DLBCL is a highly heterogeneous and invasive lymphoma with various disease sites, immune-phenotypes, and clinical prognosis. Under a microscope, abnormal large B lymphocytes can be found and the widely distributed. The cell body of abnormal B lymphocytes was usually more than twice that of normal lymphocytes (Chinese and Society of Hematology et al., 2013).

FISH is a gold standard for detecting DHL. However, FISH is difficult to perform and takes long time to operate. Besides, the reagents are expensive. In contrast, it is convenient, rapid and low cost to detect the protein expression of corresponding genes by IHC. Therefore, IHC is commonly used in clinics to detect C-MYC, BCL-2, and BCL-6 protein expression in DLBCL. Green et al. (2012b) reported that in DLBCL, there was no significant difference in survival rate between DEL and DHL, suggesting that DEL has similar biological behaviors as DHL, and that “double expression” is also associated with poor prognosis of lymphoma. Swerdlow (2014) believe that although 70–80% of DHL or THL also shows “double expression,” DEL is not equivalent to and is more common than DHL, accounting for 18–33% of DLBCL (Green et al., 2012b; Valera et al., 2013), and only < 20% of patients in DEL showed DHL (Green et al., 2012b).

The positive rate of C-MYC protein expression in this study was 47.6% (20/42), among which GCB type was 50% (8/16) and non-GCB type was 46.15% (12/26) according to the Hans typing method, and there was no significant difference between these two types. Among the 20 cases with positive C-MYC protein expression, males were 59.69% (15/26) and females were 31.25% (5/16); 51.52% (17/33) of patients were ≥50 years old and 33.33% (3/9) were < 50 years old. Statistical analysis showed that, there was no significant difference in the expression of C-MYC protein related to age or gender of patients. These results were consistent with previous studies, in which the detection rate of C-MYC gene translocation in DLBCL is 2–16% (Smith et al., 2010; Aukema et al., 2011; Gouveia et al., 2012). In DLBCL, abnormalities in the C-MYC gene mainly include translocation and amplification, often occurred in the context of complex karyotypes, and have aggressive clinical behavior.

### Biological Characteristics of C-MYC Gene Abnormality in DLBCL

Although immunohistochemical detection of C-MYC expression is not equivalent to C-MYC translocation, studies have shown that the expression of C-MYC in invasive B-cell lymphoma is associated with genetic abnormalities (Chisholm et al., 2015). Kluk et al. (2012) have shown that, when C-MYC protein expression ≥50%, the DLBCL can be identified as having the rearrangement of C-MYC gene. Green et al. proposed that if C-MYC protein expression ≥70%, the DLBCL is predicted to have translocation of C-MYC gene (Green et al., 2012a).

In addition to translocation, C-MYC gene abnormalities include amplification and mutation (Gurel et al., 2008; Leucci et al., 2008; Ruzinova et al., 2010). In the 42 cases of DLBCL in this study, only one case of C-MYC amplification (2.38%) was detected. At present, there are few reports on C-MYC amplification. Previous studies had suggested that DLBCL patients with C-MYC amplification have a relatively poor prognosis (Mossafa et al., 2006; Yoon et al., 2008; Stasik et al., 2010). The amplification rate of C-MYC gene by Stasik et al. (2010) was 38% (18/47), and C-MYC amplification was correlated with its mRNA expression. However, a report from South Korea (Yoon et al., 2008) showed a C-MYC amplification rate of 7.1% (11/156), and multiple factors have suggested that C-MYC amplification is an independent prognostic factor for poor prognosis of DLBCL.

### Biological Characteristics of BCL-2 Gene Abnormality in DLBCL

BCL-2 is detectable in approximately 50% of DLBCL and 75% of high-grade B-cell lymphomas, whose effect is to inhibit cell apoptosis and promote cell proliferation, which interacts with the action of C-MYC. BCL-2 also enhances the role of other oncogenes and induces lymphoma. BCL-2 protein accelerates the growth of lymphoma and promotes the resistance of tumor cells to chemical drugs.

The positive rate of BCL-2 protein expression in this study was 85.71% (36/42), and according to Hans typing method, 75% (12/16) of the cases were GCB and 92.31% (24/26) were non-GCB type. There was no significant difference between the types. Among the 36 cases with positive BCL-2 protein expression, males were 80.77% (21/26) and females were 93.75% (15/16), ≥50 years old were 81.82% (27/33); and < 50 years old were 100% (9/9). Statistically, there was no significant difference in the distribution of BCL-2 protein expression in age and gender.

Previous studies (Iqbal et al., 2004, 2006) reported that the incidence of BCL-2 translocation was 20–30%, and in the 42 cases of DLBCL in this study, 18 cases (42.86%) of BCL-2 were detected. One studies (Chen et al., 2010) showed that BCL-2 translocation mainly occurred in GCB type. Among the 18 cases of BCL-2 translocation that we have obtained, 7 cases were GCB type (7/16, 43.75%) and 11 cases were non-GCB type (11/26, 42.31%). Eighteen cases of BCL-2 translocation were positive for BCL-2 protein expression, while BCL-2 translocation did not occur in BCL-2 protein negative cases, suggesting that BCL-2 protein expression is prominently correlated with BCL-2 translocation.

### Biological Characteristics of BCL-6 Gene Abnormality in DLBCL

BCL-6 is a nuclear transcriptional repressor. It was reported that BCL-6 gene mutation and chromosomal translocation are the basis of tumorigenesis. Abnormal expression of BCL-6 can directly regulate cell differentiation, proliferation and apoptosis to promote tumor growth and differentiation (Ci et al., 2008). BCL-6 can affect DLBCL by regulating B cell activation, differentiation, cell cycle, and apoptosis (Polo et al., 2004). In the study of Basso and Dalla Favera (Basso and Dalla-Favera, 2012), BCL-6 regulates the follicular germinal center response and inhibits C-MYC protein and BCL-2 protein expression in normal GCB cells. In addition, BCL-6 gene also regulates tumor growth by regulating the expression of other genes, such as signal transducers and activators of transcription (STAT) and B lymphocyte induced maturation protein-1 (Blimp-1) and others (Kusam et al., 2004).

The positive rate of BCL-6 protein expression in this study was 85.71% (19/42), among which, GCB type was 43.75% (7/16), non-GCB type was 46.15% (12/26), and the detection rate of BCL-6 translocation was 14.29% (6/42). All 6 cases of BCL-6 translocation DLBCL have shown positive expression of BCL-6 protein. There was a correlation between BCL-6 gene translocation and protein expression in patients. In patients with negative BCL-6 protein expression, there was no BCL-6 translocation found. In this study, 6 DLBCL patients with BCL-6 translocation were males ≥50 years old, suggesting that DLBCL patients in middle-aged men have a relatively high BCL-6 translocation rate. Therefore, for patients with positive BCL-6 protein expression, there is a high probability of BCL-6 translocation.

### The Proliferation Index Ki-67 and Abnormalities of C-MYC, BCL-2, and BCL-6 Genes

Studies (Mationg-Kalaw et al., 2012) have reported that when the proliferation index Ki-67 is ≥90%, the sensitivity of detecting DHL/THL in invasive B-cell lymphoma was 0.54; when Ki-67≥75%, it was 77%. Another study (Landsburg et al., 2014) set the cutoff value of Ki-67 to 80% and found no difference between DHL and non-DHL. In this study, we set Ki-67 with two cut-off values: ≥80 and ≥90%, and perform statistical analysis with C-MYC, BCL-2, BCL-6 translocation, and amplification. We found similar results with the previous studies (Mationg-Kalaw et al., 2012; Landsburg et al., 2014). Therefore, high Ki-67 proliferative index indicated a higher degree of malignancy in patients with C-MYC translocation in DLBCL.

### DHL/THL and DEL/TEL

The poor prognosis caused by C-MYC translocation is mainly due to parallel BCL-2 or BCL-6 translocation (Johnson et al., 2009; Pedersen et al., 2012; Pillai et al., 2013). Studies have shown that the presence of C-MYC, BCL-2, or BCL-6 and TP53 gene alterations in DHL is important for pathogenesis and a poor prognosis of DHL (Aukema et al., 2011). Further studies (Gebauer et al., 2015) showed that there is a difference between C-MYC/BCL-2 translocation and C-MYC/BCL-6 translocation in DHL, and DHL of C-MYC/BCL-2 translocation shows frequent TP53 mutation, which rarely occurs in DHL of C-MYC/BCL-6 translocation.

Taken together, our results suggest that C-MYC, BCL-2, and BCL-6 gene translocations are correlated with their protein expression in DLBCL. Therefore, immunohistochemical staining of C-MYC, BCL-2, and BCL-6 proteins could be used for helping diagnosis and prognosis of DLBCL.

Because our sample sizes were small and our methods did not encompasses all gene abnormalities, future study with a big sample size will be merited.

## Ethics Statement

This study was approved the Ethics committee of Weifang People's Hospital.

## Author Contributions

YZ and WF conceived and designed the study. YZ, HW, and HY reviewed cases. CR and NZ collected cases. SG and WF performed statistical analyses. CR, WF, and QH performed experiments. YZ and WF wrote the manuscript. All authors read and revised the manuscript.

### Conflict of Interest Statement

The authors declare that the research was conducted in the absence of any commercial or financial relationships that could be construed as a potential conflict of interest.

## References

[B1] Arber D. A. Orazi A. Hasserjian R. Thiele J. Borowitz M. J. Le Beau M. M.. (2016). The 2016 revision to the World Health Organization classification of myeloid neoplasms and acute leukemia. Blood 127, 2391–2405. 10.1182/blood-2016-03-643544 27069254

[B2] Aukema S. M. Siebert R. Schuuring E. van Imhoff G. W. Kluin-Nelemans H. C. Boerma E. J.. (2011). Double-hit B-cell lymphomas. Blood 117, 2319–2331. 10.1182/blood-2010-09-297879 21119107

[B3] Basso K. Dalla-Favera R. (2012). Roles of BCL6 in normal and transformed germinal center B cells. Immunol. Rev. 247, 172–183. 10.1111/j.1600-065X.2012.01112.x 22500840

[B4] Campo E. Swerdlow S. H. Harris N. L. Pileri S. Stein H. Jaffe E. S. (2011). The 2008 WHO classification of lymphoid neoplasms and beyond: evolving concepts and practical applications. Blood 117, 5019–5032. 10.1182/blood-2011-01-293050 21300984 PMC3109529

[B5] Chen Y. Han T. Iqbal J. Irons R. Chan W. C. Zhu X.. (2010). Diffuse large B-cell lymphoma in Chinese patients. Am. J. Clin. Pathol. 133, 305–313. 10.1309/AJCP4H6ADGYDZMOA 20093241

[B6] Chinese and Society of Hematology Chinese Medical Association, Chinese Society of Lymphoma, and Chinese Anti-cancer Association (2013). Guidelines for the diagnosis and treatment of China's diffuse large B cell lymphoma by the Hematology Branch of Chinese Medical Association, China Anti-Cancer Association Lymphoma Professional Committee (2013 edition). Chin. J. Hematol. 34, 816–819. 10.3760/cma.j.issn.0253-2727.2013.09.019

[B7] Chisholm K. M. Bangs C. D. Bacchi C. E. Molina-Kirsch H. Cherry A. Natkunam Y. (2015). Expression profiles of MYC protein and MYC gene rearrangement in lymphomas. Am. J. Surg. Pathol. 39, 294–303. 10.1097/PAS.0000000000000365 25581730

[B8] Chung H. J. Levens D. (2005). *c-myc* expression: keep the noise down. Molecules Cells 20, 157–166. 16267388

[B9] Ci W. Polo J. M. Melnick A. (2008). B-cell lymphoma 6 and themolecular pathogenesis of diffuse large B-cell lymphoma. Curr. Opin. Hematol. 15, 381–390. 10.1097/MOH.0b013e328302c7df 18536578 PMC2748732

[B10] Gebauer N. Bernard V. Gebauer W. Thorns C. Feller A. C. Merz H. (2015). TP53 mutations are frequent events in double-hit B-cell lymphomas with MYC and BCL2 but not MYC and BCL6 translocations. Leuk. Lymphoma 56, 179–185. 10.3109/10428194.2014.907896 24679006

[B11] Gouveia G. R. Siqueira S. A. Pereira J. (2012). Pathophysiology and molecular aspects of diffuse large B-cell lymphoma. Rev. Bras. Hematol. Hemoter. 34, 447–451. 10.5581/1516-8484.20120111 23323070 PMC3545433

[B12] Green T. M. Nielsen O. de Stricker K. Xu-Monette Z. Y. Young K. H. Moller M. B. (2012a). High levels of nuclear MYC protein predict the presence of MYC rearrangement in diffuse large B-cell lymphoma. Am. J. Surg. Pathol. 36, 612–619. 10.1097/PAS.0b013e318244e2ba 22314191

[B13] Green T. M. Young K. H. Visco C. Xu-Monette Z. Y. Orazi A. Go R. S.. (2012b). Immunohistochemical double-hit score is a strong predictor of outcome in patients with diffuse large B-cell lymphoma treated with rituximab plus cyclophosphamide, doxorubicin, vincristine, and prednisone. J. Clin. Oncol. 30, 3460–3467. 10.1200/JCO.2011.41.4342 22665537

[B14] Gurel B. Iwata T. Koh C. M. Jenkins R. B. Lan F. Van Dang C.. (2008). Nuclear MYC protein overexpression is an early alteration in human prostate carcinogenesis. Mod. Pathol. 21, 1156–1167. 10.1038/modpathol.2008.111 18567993 PMC3170853

[B15] Hans C. P. Weisenburger D. D. Greiner T. C. Gascoyne R. D. Delabie J. Ott G.. (2004). Confirmation of the molecular classification of diffuse large B-cell lymphoma by immunohistochemistry using a tissue microarray. Blood 103, 275–282. 10.1182/blood-2003-05-1545 14504078

[B16] Horn H. Ziepert M. Becher C. Barth T. F. Bernd H. W. Feller A. C. (2013). MYC status in concert with BCL-2 and BCL-6 expression predicts outcome in diffuse large B-cell lymphoma. Blood 121, 2253–2263. 10.1182/blood-2012-06-435842 23335369

[B17] Iqbal J. Neppalli V. T. Wright G. Dave B. J. Horsman D. E. Rosenwald A. (2006). BCL-2 expression is a prognostic marker for the activated B-cell-like type of diffuse large B-cell lymphoma. J. Clin. Oncol. 24, 961–968. 10.1200/JCO.2005.03.4264 16418494

[B18] Iqbal J. Sanger W. G. Horsman D. E. Rosenwald A. Pickering D. L. Dave B. (2004). BCL-2 translocation defines a unique tumor subset within the germinal center B-cell-like diffuse large B-cell lymphoma. Am. J. Pathol. 165, 159–166. 10.1016/S0002-9440(10)63284-1 15215171 PMC1618550

[B19] Johnson N. A. Savage K. J. Ludkovski O. Ben-Neriah S. Woods R. Steidl C.. (2009). Lymphomas with concurrent BCL2 and MYC translocations: the critical factors associated with survival. Blood 114, 2273–2279. 10.1182/blood-2009-03-212191 19597184 PMC2745846

[B20] Kluk M. J. Chapuy B. Sinha P. Roy A. Dal Cin P. Neuberg D. S.. (2012). Immunohistochemical detection of MYC-driven diffuse large B-cell lymphomas. PLoS ONE 7:e33813. 10.1371/journal.pone.0033813 22511926 PMC3325231

[B21] Kusam S. Vasanwala F. H. Dent A. L. (2004). Transcriptional repressor BCL-6 immortalizes germinal center-like B cells in the absence of p53 function. Oncogene 23, 839–844. 10.1038/sj.onc.1207065 14737119

[B22] Landsburg D. J. Nasta S. D. Svoboda J. Morrissette J. J. Schuster S. J. (2014). ‘Double-Hit' cytogenetic status may not be predicted by baseline clinicopathological characteristics and is highly associated with overall survival in B cell lymphoma patients. Br. J. Haematol. 166, 369–374. 10.1111/bjh.12901 24761809

[B23] Leucci E. M. Cocco A. Onnis A. De Falco G. van Cleef P. Bellan C.. (2008). MYC translocation-negative classical Burkitt lymphoma cases: an alternative pathogenetic mechanism involving miRNA deregulation. J. Pathol. 216, 440–450. 10.1002/path.2410 18802929

[B24] Li X. Li G. Gao Z. (2012). Chinese lymphoma pathology research collaboration group. Chinese lymphoma subtype distribution: 10002 cases of multicenter cases in China. Diagnostic Theory Practice 11, 111–115. 10.3969/j.issn.1671-2870.2012.02.006

[B25] Mationg-Kalaw E. Tan L. H. Tay K. Lim S. T. Tang T. Lee Y. Y.. (2012). Does the proliferation fraction help identify mature B cell lymphomas with double and triple-hit translocations. Histopathology 61, 1214–1218. 10.1111/j.1365-2559.2012.04351.x 23171357

[B26] Mossafa H. Damotte D. Jenabian A. Delarue R. Vincenneau A. Amouroux I. (2006). Non-Hodgkin's lymphomas with Burkitt-like cells are associated with C-MYC amplification and poor prognosis. Leuk. Lymphoma 547, 1885–1893. 10.1080/10428190600687547 17065002

[B27] Pedersen M. O. Gang A. O. Poulsen T. S. Knudsen H. Lauritzen A. F. Nielsen S. L.. (2012). Double-hit BCL2/MYC translocations in a consecutive cohort of patients with large B-cell lymphoma-a single centre's experience. Eur. J. Haematol. 89, 63–71. 10.1111/j.1600-0609.2012.01787.x 22510149

[B28] Pillai R. K. Sathanoori M. Van Oss S. B. Swerdlow S. H. (2013). Double-hit B-cell lymphomas with BCL6 and MYC translocations are aggressive, frequently extranodal lymphomas distinct from BCL2 double-hit B-cell lymphomas. Am. J. Surg. Pathol. 37, 323–332. 10.1097/PAS.0b013e31826cebad 23348205

[B29] Polo J. M. Dell'Oso T. Ranuncolo S. M. Cerchietti L. Beck D. Da Silva G. F. (2004). Specific peptideinterference reveals BCL6 transcriptional and oncogenic mechanisms in b-cell lymphoma cells. Nat Med. 10, 1329–1335. 10.1038/nm1134 15531890

[B30] Ruzinova M. B. Caron T. Rodig S. J. (2010). Altered subcellular localization of C-MYC protein identifies aggressive B-cell lymphomas harboring a C-MYC translocation. Am. J. Surg. Pathol. 34, 882–891. 10.1097/PAS.0b013e3181db83af 20442643

[B31] Smith S. M. Anastasi J. Cohen K. S. Godley L. A. (2010). The impact of MYC expression in lymphoma biology: beyond Burkitt lymphoma. Blood Cells Mol. Dis. 45, 317–323. 10.1016/j.bcmd.2010.08.002 20817505

[B32] Stasik C. J. Nitta H. Zhang W. Mosher C. H. Cook J. R. Tubbs R. R.. (2010). Increased MYC gene copy number correlates with increased mRNA levels in diffuse large B-cell lymphoma. Haematologica 95, 597–603. 10.3324/haematol.2009.012864 20378577 PMC2857189

[B33] Swerdlow S. H. (2014). Diagnosis of 'double hit' diffuse large B-cell lymphoma, and B-cell lymphoma, unclassifiable, with features intermediate between DLBCL, and Burkitt lymphoma: when, and how, FISH versus IHC. Hematology Am. Soc. Hematol. Educ. Program 2014, 90–99. 10.1182/asheducation-2014.1.90 25696840

[B34] Valera A. López-Guillermo A. Cardesa-Salzmann T. Climent F. González-Barca E. Mercadal S.. (2013). MYC protein expression and genetic alterations have prognostic impact in patients with diffuse large B-cell lymphoma treated with immunochemotherapy. Haematologica 98, 1554–1562. 10.3324/haematol.2013.086173 23716551 PMC3789460

[B35] Yoon S. O. Jeon Y. K. Paik J. H. Kim W. Y. Kim Y. A. Kim J. E.. (2008). MYC translocation and an increased copy number predict poor prognosis in adult diffuse large B-cell lymphoma (DLBCL), especially in germinal centre-like B cell (GCB) type. Histopathology 53, 205–217. 10.1111/j.1365-2559.2008.03076.x 18752503

